# Comparative transcriptomic analysis of *Gardnerella vaginalis* biofilms vs. planktonic cultures using RNA-seq

**DOI:** 10.1038/s41522-017-0012-7

**Published:** 2017-02-02

**Authors:** Joana Castro, Angela França, Katie R. Bradwell, Myrna G. Serrano, Kimberly K. Jefferson, Nuno Cerca

**Affiliations:** 1 Centre of Biological Engineering (CEB), Laboratory of Research in Biofilms Rosário Oliveira (LIBRO), University of Minho, Campus de Gualtar, 4710-057 Braga, Portugal; 2 Instituto de Ciências Biomédicas Abel Salazar (ICBAS), University of Porto, Rua de Jorge Viterbo Ferreira 228, 4050-313 Porto, Portugal; 3 Department of Microbiology and Immunology, Virginia Commonwealth University, Richmond, VA 23298-0678c USA; 4 Center for the Study of Biological Complexity, Virginia Commonwealth University, Richmond, VA 23284 USA

## Abstract

Bacterial vaginosis is the most common gynecological disorder affecting women of reproductive age. Bacterial vaginosis is frequently associated with the development of a *Gardnerella vaginalis* biofilm. Recent data indicates that *G. vaginalis* biofilms are more tolerant to antibiotics and are able to incorporate other bacterial vaginosis -associated species, yielding a multi-species biofilm. However, despite its apparent role in bacterial vaginosis, little is known regarding the molecular determinants involved in biofilm formation by *G. vaginalis*. To gain insight into the role of *G. vaginalis* in the pathogenesis of bacterial vaginosis*,* we carried out comparative transcriptomic analysis between planktonic and biofilm phenotypes, using RNA-sequencing. Significant differences were found in the expression levels of 815 genes. A detailed analysis of the results obtained was performed based on direct and functional gene interactions. Similar to other bacterial species, expression of genes involved in antimicrobial resistance were elevated in biofilm cells. In addition, our data indicate that *G. vaginalis* biofilms assume a characteristic response to stress and starvation conditions. The abundance of transcripts encoding proteins involved in glucose and carbon metabolism was reduced in biofilms. Surprisingly, transcript levels of vaginolysin were reduced in biofilms relative to planktonic cultures. Overall, our data revealed that gene-regulated processes in *G. vaginalis* biofilms resulted in a protected form of bacterial growth, characterized by low metabolic activity. This phenotype may contribute towards the chronic and recurrent nature of bacterial vaginosis. This suggests that *G. vaginalis* is capable of drastically adjusting its phenotype through an extensive change of gene expression.

## Vaginal infection: Gene activity protects biofilms

Altered gene activity may promote chronic and recurrent infections in the most common gynecological disorder in women of reproductive age. Bacterial vaginosis (BV) frequently involves the formation of biofilms by *Gardnerella vaginalis* bacteria. Nuno Cerca and colleagues at the University of Minho in Portugal, with co-workers elsewhere in Portugal and in the USA, studied changes in gene activity in BV biofilms. Their results linked differences in the activity of 815 genes to an ability of the bacteria to enter a protected form of growth when producing biofilms. The differences included increased activity of specific genes associated with antibiotic resistance. This insight into the bacterial ability to undergo drastic changes in gene activity could guide work to understand chronic and recurrent BV. It may also lead to new ways to treat this common and troublesome condition.

## Introduction

Bacterial vaginosis (BV) is the most prevalent vaginal condition in women of reproductive age and can cause several problems, such as preterm birth, endometritis, and increased risk of acquisition and transmission of sexual transmitted diseases.^1^ Examination of vaginal biopsy specimens has demonstrated that most cases of BV are characterized by the adherence of a bacterial biofilm to the vaginal epithelium, and that *Gardnerella vaginalis* is the predominant species of the biofilm mass.^2^ However, *G. vaginalis* colonization does not always lead to BV.^3^ Biofilm formation represents a protected mode of growth that allows cells to survive in the acidic vaginal environment.^4^ *G. vaginalis* can also adopt a planktonic phenotype that differs greatly from biofilm lifestyle.^5^ It is postulated that a biofilm provides an ecological advantage over planktonic bacteria.^6^

Importantly, biofilm infections are particularly problematic because sessile bacteria are generally much more tolerant to antibiotics than planktonic cells.^6^ Evidence suggests that biofilm formation contributes significantly to BV treatment failure and high recurrence rates.^7,8^ Targeting virulence factors represents a new paradigm in the development of new and effective treatments to prevent and treat biofilm-associated infections.^9^ Therefore, a better understanding of BV-associated *G. vaginalis* biofilm physiology and virulence is needed to understand the high persistence and resistance of biofilm cells.

The purpose of our study was, therefore, to identify the major transcriptomic features of BV-associated *G. vaginalis* biofilms, as compared to their planktonic counterparts, using high-throughput RNA-sequencing (RNA-seq).Transcriptomic comparisons between biofilm and planktonic cultures that have been carried out for *Staphylococcus aureus*,^10^ *Staphylococcus epidermidis*,^11^ *Streptococcus mutans,* ^12^ and *Streptococcus pneumonia*,^13^ indicate that gene-regulated processes in the biofilm led to a protective mode of growth by developing an effective cellular response to stress and decreasing metabolic activity.

Herein, we sequenced the transcriptome of BV-associated *G. vaginalis* biofilms and planktonic cultures and used a data analysis approach based on direct and functional gene interactions, namely gene set enrichment and cluster analysis.

## Results

### Transcriptome analysis

A total of 561,302 (planktonic phenotype) and 311,643 (biofilm phenotype) sequencing reads were obtained for the complementary DNA (cDNA) libraries. Before trimming the raw data, we identified the genes, with the reads per kilobase per million (RPKM) above 1.00, expressed in each condition. We only detected three genes uniquely expressed in biofilm cells, whereas 11 genes were found uniquely in planktonic cells. However, the majority of gene transcripts that were only detected in planktonic or biofilm cells, encoded uncharacterized proteins or transfer RNA, as shown in the Supplementary Material (Table S1).

Our data indicated that within the 1045 genes that were transcribed in both conditions, 815 (78%) were differentially expressed between planktonic and biofilm cells. For downstream analysis, only genes with fold-changes above two were considered. Transcript levels of 309 (30%) genes were elevated, whereas 36 (3%) were reduced in biofilms. Among the transcripts that were more abundant in biofilms, 78 encoded hypothetical proteins. In an effort to find homology with known proteins, we performed a BLAST analysis, a search in the Pfam database (version 29.0) for Pfam domains^14^ and used the PSORTb program (v.3.0)^15^ to predict their subcellular localization. The results are shown in Table S2. Interestingly, 53% of these proteins might have cytoplasmic membrane localization, suggesting that part of these proteins could have a transporter function.

**Fig. 1:**
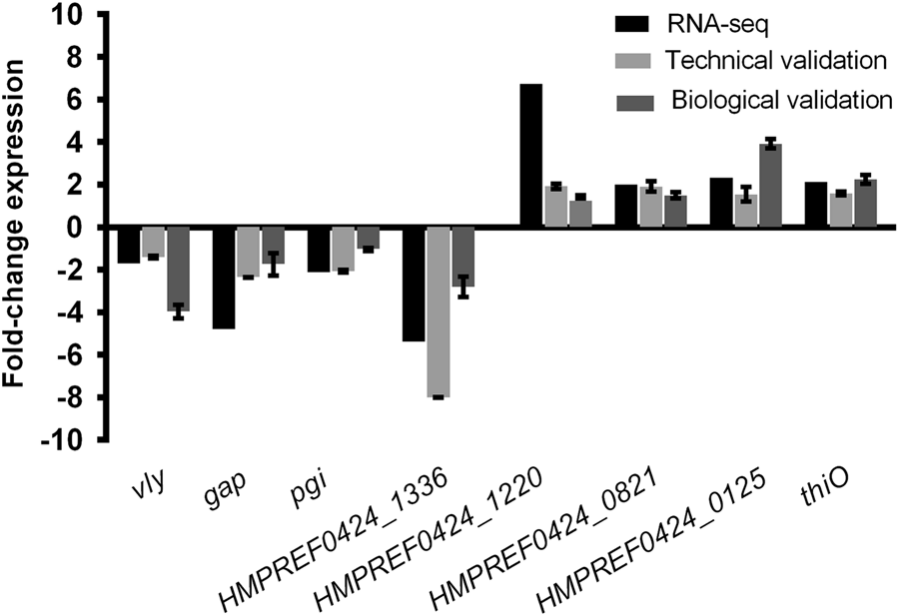
qPCR validation of the transcription of differentially expressed genes randomly selected. Technical validation means that we used the same total RNA utilized for libraries construction. Biological validation means that we used new total RNA obtained from independent experiments performed under same biological conditions. The data indicate the fold-change expression of genes in *G. vaginalis* biofilms cells compared to planktonic cells. For qPCR experiments, the bars represent the mean and the error bars the standard error of the mean (mean ± SEM)

In order to confirm the results obtained by RNA-seq, transcripts detected in greater or lesser abundance in biofilms were randomly selected and their relative levels quantified by quantitative PCR (qPCR). Both RNA used for cDNA libraries construction (technical validation) and RNA obtained by performing new experiments (biological validation) were used for validation. As can be seen in Fig. 1, the same trend was observed in all measurements (qPCR and RNA-seq).

### Enrichment analysis of genes with increased and decreased transcription

**Fig. 2:**
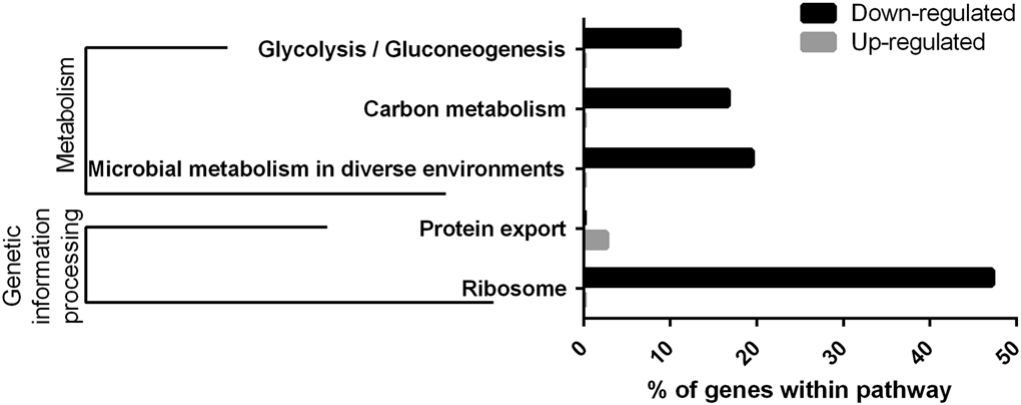
KEGG pathways found significantly enriched (*p* < 0.05) within the genes with increased and decreased transcription in biofilm cells

GO annotation, placement of genes on Kyoto Encyclopedia of Genes and Genomes (KEGG) pathways, and enrichment analysis of the genes with down and upregulated transcription was accomplished using STRING.^16^ Significant enrichment was only found (*p* < 0.05, false discovery rate (FDR)-corrected) in KEGG pathways (Fig. 2). As could be expected, classes associated with metabolism were found significantly enriched among the genes with decreased transcription, suggesting that biofilm cells were less metabolically active than planktonic cells. Conversely, protein export was found to be enriched among transcripts that were elevated in biofilm cells.

### Cluster analysis

**Fig. 3:**
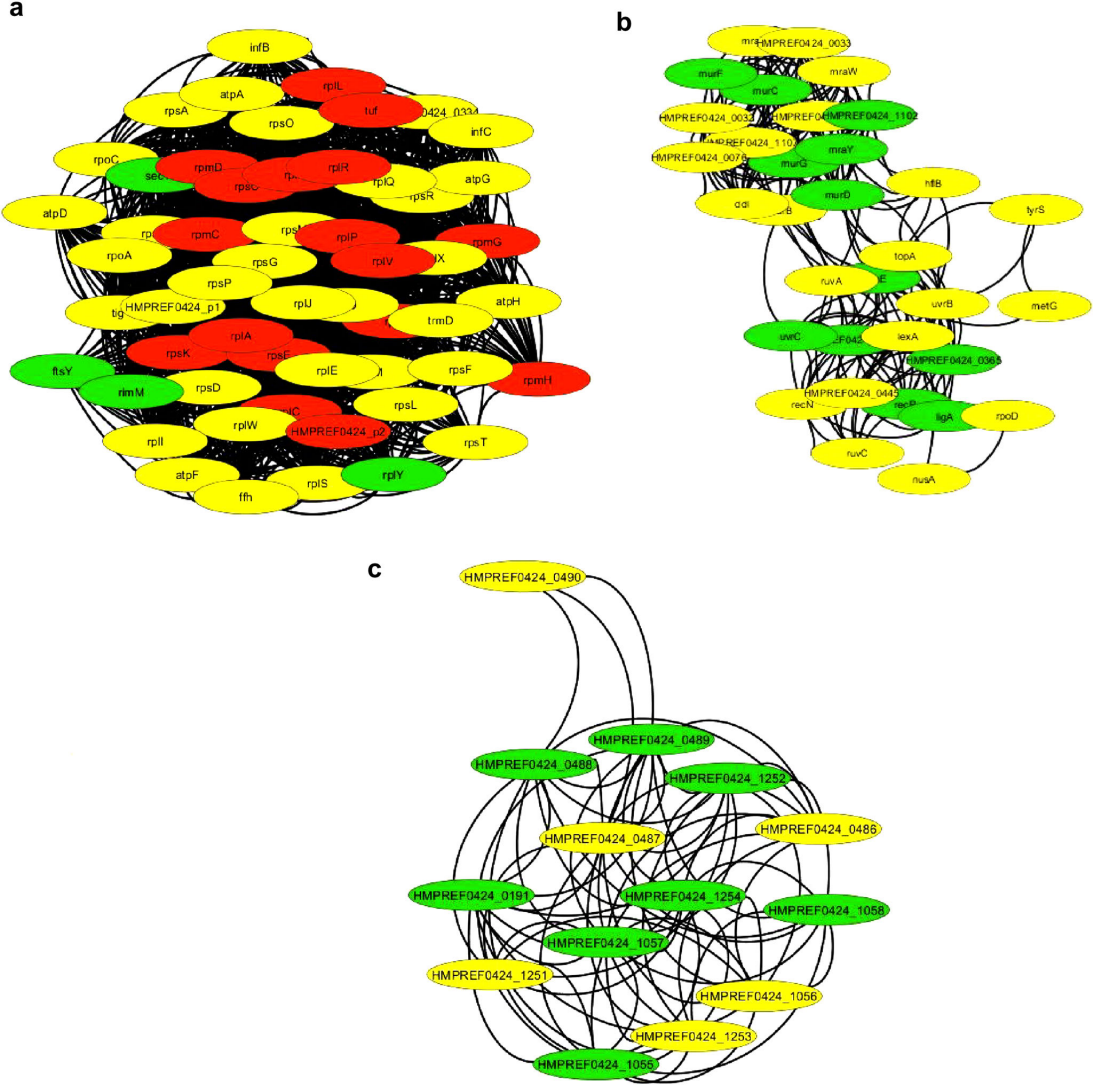
Clusters generated by the MCODE plugin in Cytoscape. *Red*, *green*, and *yellow circles* represent fold-change values under −2, above 2, and between −2 and 2, respectively. Biological process indicates enrichment (*p* < 0.05) in translation and metabolic process (**a**); cell-wall biosynthesis biogenesis and mismatch repair (**b**); and KEGG indicates β-Lactamase resistance (**c**)

Gene clustering analysis was based on direct and functional gene interactions using Cytoscape.^17^ Cytoscape was used to create a gene interaction network including all differently expressed genes and neighbors, yielding a total of 764 nodes and 7685 edges (complete gene network of differently expressed genes is shown in the Supplementary Material Fig. S1). Among the differently expressed genes, we found 22 clusters. Significant enrichment was found (*p* < 0.05, FDR-corrected) in biological processes or KEGG pathways associated with translation and metabolic process (Fig. 3a), cell-wall biogenesis and mismatch repair (Fig. 3b), and antimicrobial resistance (Fig. 3c).

### The top 10 most significantly down or upregulated genes in biofilms

Table 1 lists the 10 transcripts with the greatest increase and the 10 with the greatest decrease in biofilm cells. Among the transcript decreased, we found ribosomal proteins suggesting that biofilms had decreased level of translation. Furthermore, BV-associated *G. vaginalis* biofilm cells showed decreased transcript levels of genes encoding several factors involved in energy production, such as *HMPREF0424_1336,* a gene encoding primary receptors for chemotaxis and transport of many sugar based solutes. In addition, the expression levels of genes associated with glucose metabolic pathways were also lower in biofilms cells, namely *gap*, that also has a role in oxidoreductase activity, and *gpmA,* that displays an important role in a subpathway of the glycolysis pathway (glycolysis/gluconeogenesis pathway of *G. vaginalis* 409-05 is shown in the Supplementary Material Fig. S2), which itself is part of carbohydrate degradation. Taken together, these results imply that *G. vaginalis* biofilm cells are characterized by the reduction of basic cell processes(translation) and metabolism (glycolysis and carbon metabolism).

**Table 1:** List of the 10 genes with lowest and highest fold-change values among the differentially expressed genes in *G. vaginalis* cultured under biofilm vs. planktonic conditions

	Gene	Definition	Fold-change
			(Biofilm vs. planktonic cells)
Rank	Downregulated		
1	*HMPREF0424_0046*	50S ribosomal protein L34	−21.93
2	*HMPREF0424_0269*	50S ribosomal protein L30	−8.99
3	*HMPREF0424_0429 (xseA)*	Exodeoxyribonuclease VII large subunit	−6.73
4	*HMPREF0424_0260*	30S ribosomal protein S3	−5.43
5	*HMPREF0424_1336*	Periplasmic-binding protein and sugar-binding domain of the LacI family protein	−5.39
6	*HMPREF0424_0259*	50S ribosomal protein L22	−5.36
7	*HMPREF0424_0258*	50S ribosomal protein L2	−4.93
8	*HMPREF0424_0471 (gap)*	Glyceraldehyde 3-phosphate dehydrogenase domain-containing protein	−4.81
9	*HMPREF0424_0276*	30S ribosomal protein S11	−3.86
10	*HMPREF0424_0394 (gpmA)*	Phosphoglyceratemutase	−3.62
Rank	Upregulated		
1	*HMPREF0424_0510*	Uncharacterized protein	14.41
2	*HMPREF0424_0563*	Pyroglutamyl-peptidase I	9.57
3	*HMPREF0424_1220*	Aminotransferase, class I/II	6.74
4	*HMPREF0424_0420*	LPXTG-motif cell wall anchor domain-containing protein	6.30
5	*HMPREF0424_0397*	Uncharacterized protein	5.76
6	*HMPREF0424_0573*	LysM domain-containing protein	5.51
7	*HMPREF0424_0943*	ComEA protein	5.15
8	*HMPREF0424_0166*	Uncharacterized protein	4.28
9	*HMPREF0424_0888*	NLPA lipoprotein	4.24
10	*HMPREF0424_0797*	Uncharacterized protein	4.12

Among the transcripts elevated in biofilm cells, we found *HMPREF0424_0563,*a gene with a molecular function related to hydrolase activity. Furthermore, in biofilm cells we found an overexpression of the *HMPREF0424_1220* gene encoding an aminotransferase involved in amino acid biosynthesis. A similar trend was reported for *Neisseria meningitides*.^18^ Interestingly, we found *HMPREF0424_0420*, a gene that encodes the LPXTG-motif cell anchor domain-containing protein, which can be involved in biofilm formation, as described in Gram-positive bacteria.^19^ Moreover, in BV-associated *G. vaginalis* biofilm cells, transcript levels of the gene *HMPREF0424_0573*, which encodes a LysM domain-containing protein possibly associated with autoaggregation of *G. vaginalis,* were also increased, similar to what was observed for *Lactobacillus reuteri* biofilms.^20^ Transcripts encoding the ComEA protein (*HMPREF0424_0943),* which is involved in DNA repair and NLPA lipoprotein (*HMPREF0424_0888* gene), which is involved in ABC transporters were also found in greater abundance in biofilms cells.

### Upregulation of the transcription of potential virulence genes in *G. vaginalis* biofilms

**Fig. 4:**
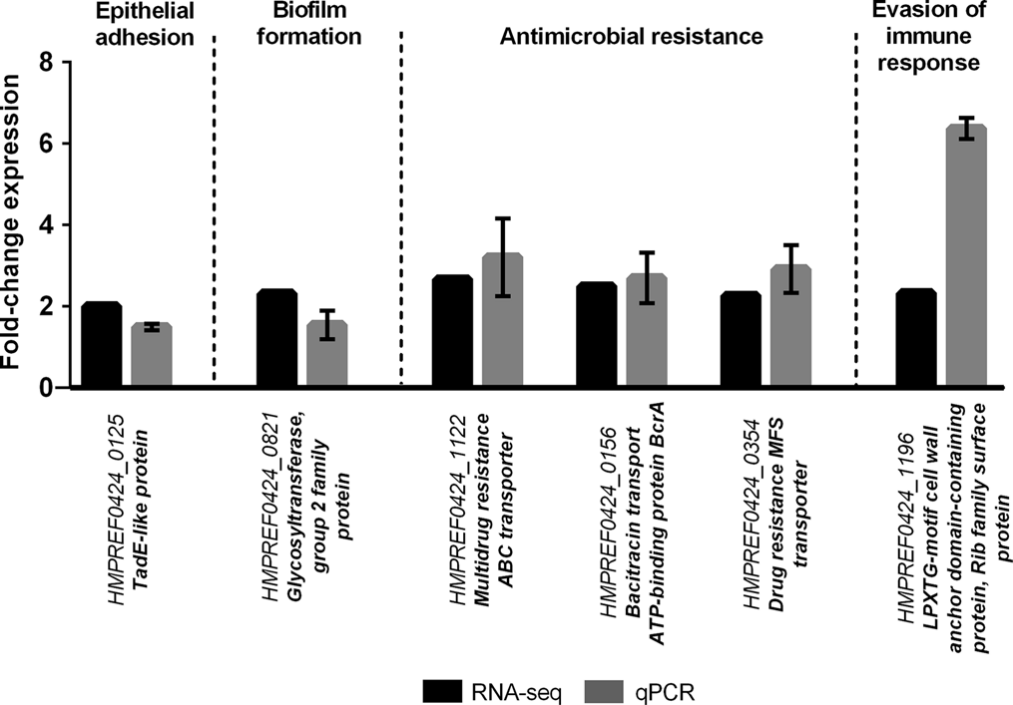
Quantification of the transcription of known virulence genes in *G. vaginalis* cultured under biofilm and planktonic conditions*.* Bars represent the mean and the error bars the standard error of the mean (mean ± SEM)

Biofilm formation by pathogenic bacteria is often associated with altered virulence. Bacterial biofilms may suppress certain virulence factors while others are activated in order to evade immune defenses, and survive challenging conditions.^21^ It was, therefore, of interest to determine the expression levels of previously annotated potential virulence genes.^22^ As can be seen in Fig. 4, we found a slight increase in the *HMPREF0424_0125* transcript, which encodes *TadE*-like protein. This might play an important role in adhesion to vaginal epithelial cells, similarly to what has been described in *Actinobacillus actinomycetemcomitans*.^23^ Furthermore, our data supported the previous hypothesis that *G. vaginalis* biofilm development is likely associated with type II glycosyltransferase.^22^ Of note, glycosyltransferases are likely to be important for the biosynthesis of exopolysaccharide which in turn is important for biofilm formation. The ability to grow as a biofilm would likely confer an increase in antibiotic tolerance and resistance to mucosal immune defenses.^24^ Herein, levels of transcripts encoding antimicrobial-specific resistance proteins belonging to efflux pump families were increased. In addition, the *HMPREF0424_1196* transcript, which encodes a Rib-protein, was elevated in biofilm cells. Rib proteins belong to the α-like protein (Alp)-family of highly repetitive surface antigens and are commonly found in Gram-positive pathogens.^25^ These proteins elicit protective immunity through their inter-strain size variability.^22^

### Differential expression of vaginolysinin BV-associated *G. vaginalis* biofilms

**Fig. 5:**
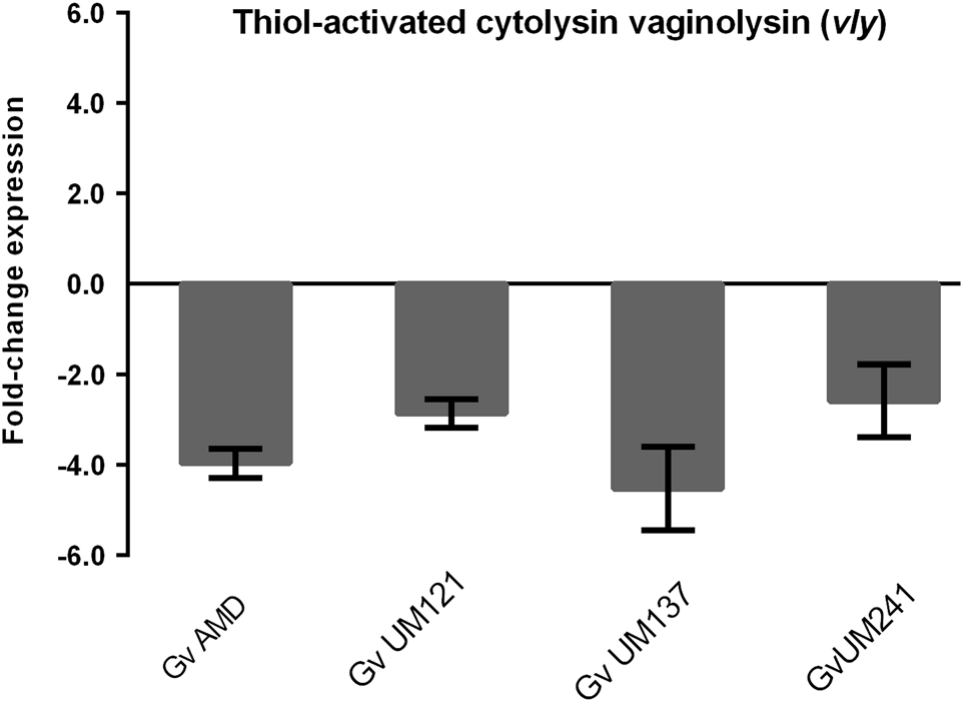
Quantification of *thiol-activated cytolysinvaginolysin* (*vly)* transcription in *G. vaginalis* strains. Bars represent the mean and the error bars the standard error of the mean (mean ± SEM)

*G. vaginalis* produces athiol-activated cholesterol-dependent cytolysin, vaginolysin (*vly*), which might induce vaginal cells lysis. Strikingly, in our experiments, the expression levels of *vly* (*HMPREF0424_0103)* were significantly lower in biofilm cells (Fig. 1). In order to determine whether this was a strain-specific variation, we evaluated *vly* gene expression, by qPCR, in three other biofilm forming isolates, which were previously characterized.^26^ Interestingly, as shown in Fig. 5, the downregulation of the transcription of *vly* was observed in all different isolates.

## Discussion

As noted elsewhere, gene expression profiles can reveal important information about the adaptation of a bacterial species to a particular environmental niche. Therefore, adaptation to a given host environment is an extremely important factor and underlies the capacity of a colonizing species or a pathogen to persist in a host.^6^ In the present study, we analyzed the transcriptome of a BV-associated *G. vaginalis* cultivated under biofilm and planktonic conditions. Our results demonstrated that more transcripts were increased in biofilm relative to planktonic cells. Importantly, our findings provide key insights into the development of biofilms and the pathogenicity of *G. vaginalis*, the predominant bacterial species isolated in women with BV.^27,28^

Here, we showed that BV-associated *G. vaginalis* biofilm cells alter their gene expression profile, namely transcript levels of genes involved in metabolism (with downregulation of genes associated with glycolysis and carbon metabolism) and translation (with downregulation of genes encoding ribosomal proteins), as also reported for other microorganisms such as *S. epidermidis*,^11^ *S. aureus,* ^10^ and *S. mutans*.^12^ In *G. vaginalis* biofilms, cell density is substantially higher than in planktonic culture.^4^ As a consequence, most biofilm cells are likely to encounter restricted availability of nutrients.^29^ Similar to what was found for *S. mutans* ^30^ and *S. pneumonia*,^13^ we also observed that the transcripts encoding ABC transporter proteins were elevated in biofilm cells. In addition, our study revealed that transcripts of genes involved in the synthesis of peptidoglycan and cell wall were also greater in biofilms. This has also been shown for *P. aeruginosa* ^31^ and *S. aureus*.^10^ It has been hypothesized that the cell envelope is a highly dynamic and active component of biofilm cells, contributing to its persistence.^10,13^ However, the reasons for the overexpression of genes involved in cell wall biogenesis require further investigation.

Notably, transcripts of other potential virulence genes, previously annotated by Yeoman and colleagues,^22^ were also more abundant in biofilm cells, with the exception of *vly*. Several studies have highlighted the role of *vly* gene in *G. vaginalis* virulence.^32,33^ The *vly* gene belongs to the cholesterol-dependent cytolysins (CDCs), a family of pore-forming toxins, which cause cytotoxicity on vaginal epithelium.^33^ Our previous findings showed that planktonic cultures of BV isolates of *G. vaginalis* expressed twofold more *vly* than planktonic cultures of non-BV isolates.^26^ Herein, we found that *vly* transcript levels were higher in planktonic than in biofilms cells. The low levels of expression of *vly* in biofilms might reflect the more chronic nature of vaginal colonization by BV-associated *G. vaginalis* and serve as a means towards preventing a host immune response. Similarly, Resch and colleagues^10^ showed that the production of various *S. aureus* toxins were significantly upregulated in planktonic rather than in biofilm cells, suggesting that toxins may not be conducive to biofilm persistence in the host.

Similar to what was observed in other microorganisms, BV-associated *G. vaginalis* biofilm phenotype might induce aquiescent mode of growth that is less sensitive to antibiotics, as the efficacy of many antibiotics relies on active cell metabolism and the cell-wall construction process.^11^ Here, we observed that efflux pumps and ABC transporters, reported as mechanisms responsible for antimicrobial resistance,^34^ were upregulated in biofilms cells. Comparable evidence for the role of efflux pumps in biofilm resistance has been found in several microorganisms such as *Pseudomonas aeruginosa*,^35^ *Escherichia coli,* ^36^ and *Candida albicans*.^37^

Taken together, these data indicated that BV-associated *G. vaginalis* changes its transcriptomic profile when growing as a biofilm. These changes are likely important for biofilm persistence and, consequently, for the virulence of this bacterium. Furthermore, the fact that *vly* is downregulated in biofilms represents an important finding, that might contribute towards a better understanding of the pathogenesis of BV. However, this study is limited by the fact that the growth medium did not contain all of the factors found in vivo, and some in vivo cues may turn on the expression of biofilm-related genes. Nevertheless, as animal models for BV are lacking, in vitro models can be very informative, and are key to furthering our understanding of virulence potential of *G. vaginalis*. In conclusion, our findings showed that the gene expression profile of BV-associated *G. vaginalis* biofilms characterizes a distinct physiologic status that may promote the chronic and recurrent nature of BV.

## Materials and methods

### Bacterial strains

*G. vaginalis* strain AMD, isolated from a woman diagnosed with BV based on Amsel criteria at VCU Women’s Health Clinic,^38^ was used for RNA-seq analysis. *G. vaginalis* strains UM121, UM137, and UM241, also isolated from women with BV based on Amsel and Nugent criteria,^26^ were used for subsequent analysis.

### Planktonic growth

Planktonic cells were grown in sBHI [Brain-heart infusion supplemented with 2% (wt/wt) gelatin (Liofilchem, Rosetodegli Abruzzi, Italy), 0.5% (wt/wt) yeast extract (Liofilchem) and 0.1% (wt/wt) soluble starch (Panreac, Barcelona, Spain)] for 24 h at 37 °C with 10% CO_2_ (Shel Lab, Cornelius, Oregon, USA).^26^ At this time, planktonic cells were still in the exponential growth phase. Thereafter, 18 mL of planktonic cells were harvested by centrifugation (20 min, 7197×*g*) and suspended in 1 mL of RNA protect [diluted 2:1 in phosphate-buffered saline (PBS); QIAGEN, Germany].

### Biofilm formation

For biofilm formation, the cell concentration of 24 h old cultures was assessed by optical density at 600 nm (Model Sunrise, Tecan, Switzerland) and was further diluted in order to obtain a final concentration of approximately 10^6^ CFU/mL. After homogenization, 200 μL of *G. vaginalis* suspensions were dispensed into each well of three 96-well flat-bottom tissue culture plates (Orange Scientific, Braine L’Alleud, Belgium). The tissue culture plates were then incubated at 37 °C in 10% CO_2_. After 24 h, the culture medium covering the biofilms was removed, replaced by fresh sBHI and allowed to grow, under the same conditions, for an additional 24 h. This time was required for this strain to develop a notable biofilm. Forty-eight hour biofilms were then washed once with 1 × PBS, scraped from the bottom of 96-well plates in sBHI and pooled together. Finally, biofilm cells were harvested by centrifugation (20 min, 7197×*g*) and suspended in 1 mL of RNA protect (as described above).

### RNA extraction

Total RNA was extracted using a combination of mechanical lysis (3.0 mm zirconium beads, Sigma-Aldrich Inc., St. Louis, MO, USA) and the columns of the RNeasy Mini kit (QIAGEN), as optimized before (França *et al*.^49^). To remove genomic DNA, TURBO DNA-free™ kit (Ambion, Austin, TX, USA) was used as indicated by the manufacturer followed by acid-phenol:chloroform:isoamyl alcohol (125:24:1) treatment. RNA integrity was determined using an Experion^TM^ automated electrophoresis system (Bio-Rad, Hercules, CA, USA), and samples with RNA Quality Indicator (RQI) above eight were selected for cDNA library preparation.

### cDNA library preparation and sequencing

cDNA libraries were constructed using the kit ScriptSeq™ Complete Kit—low input (Illumina, San Diego, WI, USA), which already includes the kit for ribosomal (rRNA) depletion: Ribo-Zero™ Kit (Bacteria)—Low Input (Illumina, Madison, WI, USA). The construction of the libraries was rigorously validated by qPCR and Hi-Sensitivity D1K TapeStation (Agilent 2200 TapeStation). Libraries were then multiplexed and sequencing data generated from paired-end reads (2 × 150 bp) using a MiSeq system (Illumina).

### RNA-sequencing data analysis

After sequencing, adapters were trimmed by MiSeq® internal software during the base calling. Quality, ambiguity and length trimming, as well as mapping to the reference genome, and normalization of gene expression were performed using CLC Genomics Workbench version 8 (MA, USA). Quality, ambiguity and length trimming were performed using the CLC genomics workbench default settings. RNA-seq reads were aligned to the reference genome of *G. vaginalis* strain 409-05 (GenBank accession number NC_013721).Gene expression was normalized using RPKM, that account for both library size and gene length, as described by Mortazavi and colleagues.^39^ To detect significant gene expression alterations, Kal’s test^40^ with FDR^41^ correction was applied. A *p*-value ≤ 0.05 was considered statistically significant. Transcripts uniquely expressed in each condition were identified using BioinfoGP.^42^ Data were deposited at Gene Expression Omnibus database (accession number GSE8012, available at: http://www.ncbi.nlm.nih.gov/geo/query/acc.cgi?acc=GSE80127).

### Biological interactions

In order to determine the function of differentially expressed genes, gene ontology (GO)^43^ and KEGG pathway^44^ assignations and enrichment analysis were performed using the Search Tool for the Retrieval of Interacting Genes/Proteins (STRING) (version 10).^16^ In addition, UniProt repository^45^ was used to determine the function of proteins that were not identified by STRING. Classes with *p*-value ≤ 0.05, FDR-adjusted, were considered statistically significant for enrichment. Further analysis was carried out using Cytoscape (version 3.2.1),^46^ in which a gene interaction network including all differentially expressed genes and neighbors created by STRING^16^ was imported. Gene clusters (regions of high connectivity) were obtained in Cytoscape with the MCODE plugin.^47^ Default parameters (score value above two and at least four nodes) were used as the cutoff criteria for network module screening. Thereafter, an enrichment analysis of clusters was performed using STRING with a threshold of *p* < 0.05, FDR-adjusted.^16^

### Quantitative PCR

In order to validate RNA-seq data, qPCR was performed to quantify the transcription of eight randomly selected genes, by using the same total RNA utilized for libraries construction (technical validation) and new total RNA obtained from independent experiments performed under the same biological conditions (biological validation). Furthermore, the gene expression profile of known virulence genes was also addressed*.* Oligonucleotide primers were designed using Primer3^48^ having *G. vaginalis* 409-05 genome as template (Table S3). The same amount of total RNA (300 ng/µL) was reverse transcribed using the RevertAid™ First Strand cDNA synthesis kit (Fermentas, Thermo Fisher Scientific, Stanford, CA, USA), as previously optimized,^49^ and random primers (NZYTech, Lisbon, Portugal) as priming strategy. The qPCR reaction was prepared by mixing together 5 µL of iQ SYBR green supermix (Bio-Rad), 2 µL of 1:800 diluted cDNA, 0.5 µL of 5 µM forward and reverse primes and water up to 10 µL. The run was performed in a CFX96^TM^ thermal cycler (Bio-Rad) with the following cycling parameters: 3 min at 95 °C, followed by 45 cycles of 10 s at 95 °C, 10 s at 60 °C, and 15 s at 72 °C. Reaction efficiency was determined by the dilution method.^50^ At 60 °C all set of primers used had the highest and more similar efficiencies. Normalized gene expression was determined by using the delta *C*_t_ method (*E* ^Δ*C*t^), a variation of the Livak method, where Δ*C*_t_ = *C*_t_ (reference gene)—*C*_t_ (target gene) and *E* stands for the reaction efficiency experimentally determined. A non-reverse transcriptase control was included in each reaction. Three biologic replicates of each condition were analyzed.

## Electronic supplementary material

Supplementary Figure 1

Supplementary Figure 2

Supplementary Tables
