# Supplementary figures and images for: Comparative transcriptomic analysis of Gardnerella vaginalis biofilms vs. planktonic cultures using RNA-seq
Source: NPJ Biofilms Microbiomes. 2017 Feb 2;3:3. doi: 10.1038/s41522-017-0012-7 (PMC5460279; doi:10.1038/s41522-017-0012-7)

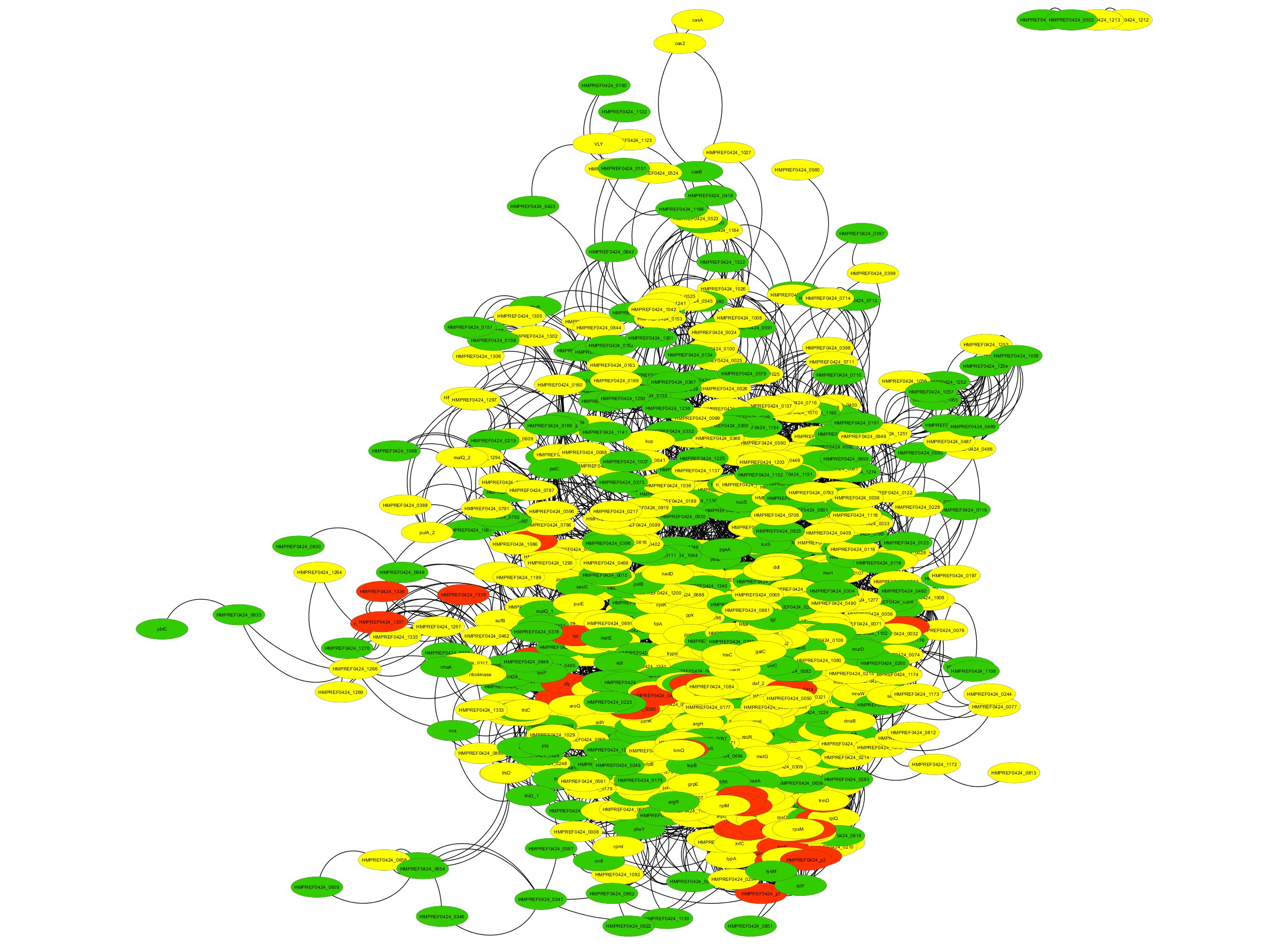

Supplement: Supplementary file 1 — Supplementary Figure 1 [file 41522_2017_12_MOESM1_ESM.tif]

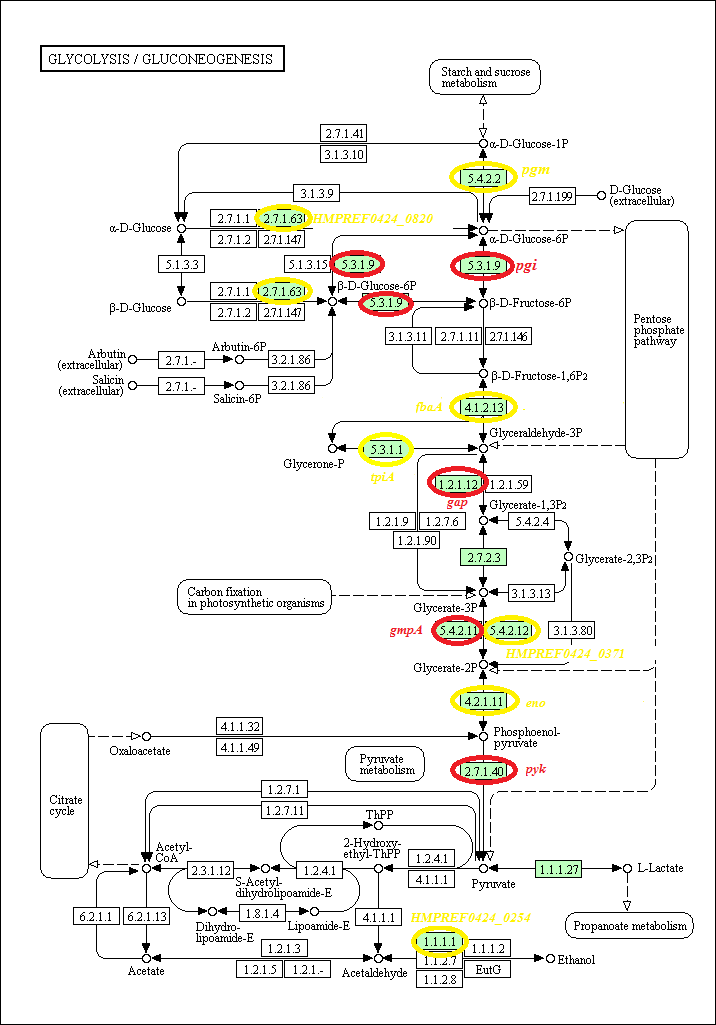

Supplement: Supplementary file 2 — Supplementary Figure 2 [file 41522_2017_12_MOESM2_ESM.tif]
