# Supplementary material for: Comparative transcriptomic analysis of Gardnerella vaginalis biofilms vs. planktonic cultures using RNA-seq
Source: NPJ Biofilms Microbiomes. 2017 Feb 2;3:3. doi: 10.1038/s41522-017-0012-7 (PMC5460279; doi:10.1038/s41522-017-0012-7)
Supplement: Supplementary file 3 — Supplementary Tables [file 41522_2017_12_MOESM3_ESM.docx]

Supplementary data

**Comparative transcriptomic analysis of *Gardnerella vaginalis* biofilms *versus* planktonic cultures using RNA-seq**

Joana Castro, Angela França, Katie R. Bradwell, Myrna G. Serrano, Kimberly K. Jefferson and Nuno Cerca*

* Corresponding author: [nunocerca@ceb.uminho.pt](mailto:nunocerca@ceb.uminho.pt)

Centre of Biological Engineering (CEB), Laboratory of Research in Biofilms Rosário Oliveira (LIBRO), University of Minho, Campus de Gualtar, 4710-057 Braga, Portugal

Tel.: +351 253604423, Fax: +351 253604429.

**Tables**

**Table S1** Lis of genes uniquely expressed in *G. vaginalis* cultured under planktonic or biofilm conditions and their known functions

| **Gene** | **Description** |
| --- | --- |
| **Planktonic unique genes** |  |
| *HMPREF0424_RS02025* | tRNA-Arg |
| *HMPREF0424_RS05465* | tRNA-Asp |
| *HMPREF0424_RS04945* | tRNA-Gln |
| *HMPREF0424_RS00345* | tRNA-Lys |
| *HMPREF0424_RS05080* | tRNA-Arg |
| *HMPREF0424_RS00030* | tRNA-Gly |
| *HMPREF0424_RS05015* | tRNA-His |
| *HMPREF0424_RS01165* | tRNA-Tyr |
| *SrpB* | Signal recognition particle RNA |
| *HMPREF0424_RS06130* | Uncharacterized protein |
| *HMPREF0424_RS02080* | Exodeoxyribonuclease 7 small subunit. Bidirectionally degrades single-stranded DNA into large acid-insoluble oligonucleotides, which are then degraded further into small acid-soluble oligonucleotides |
| **Biofilm unique genes** |  |
| *HMPREF0424_RS03150* | Uncharacterized protein |
| *HMPREF0424_RS05930* | tRNA-Trp |
| *HMPREF0424_RS02715* | Pyroglutamyl-peptidase I. Removes 5-oxoproline from various penultimate amino acid residues except L-prolin |

**Table S2** Differentially expressed genes encoding hypothetical proteins with significant *pfam* domain, including predicted localization by PSORTb and pBLAST results

| **Gene ^a^** | **Predicted localization** | **Protein family (Pfam) domain match** | **Blastp ^b^** |
| --- | --- | --- | --- |
| *HMPREF0424_0510* | Cytoplasmatic | Domain of unknown function | Hypothetical protein |
| *HMPREF0424_0397* | Cytoplasmatic membrane | Protein of unknown function | Hypothetical protein |
| *HMPREF0424_0166* | Unknown | Uncharacterized protein family | FMN-binding protein |
| *HMPREF0424_0797* | Unknown | MerR HTH family regulatory protein | MerR family transcriptional regulator |
| *HMPREF0424_0712* | Cytoplasmatic membrane | Uncharacterized protein family | Hypothetical protein |
| *HMPREF0424_1216* | Cell wall | Uncharacterized protein family | Hypothetical protein |
| *HMPREF0424_0922* | Cytoplasmatic membrane | Inner membrane component domain | Membrane protein |
| *HMPREF0424_0502* | Cytoplasmatic membrane | Uncharacterized protein family | Hypothetical protein |
| *HMPREF0424_1106* | Extracellular | Uncharacterized protein family | UDP-N-acetylmuramyl peptide synthase |
| *HMPREF0424_0796* | Cytoplasmatic membrane | Uncharacterized protein family | Hypothetical protein |
| *HMPREF0424_0123* | Unknown | Domain of unknown function: conserved EYA sequence motif | Hypothetical protein |
| *HMPREF0424_0135* | Cytoplasmatic | Protein of unknown function | Endonuclease |
| *HMPREF0424_0150* | Unknown | Uncharacterized protein family | Hypothetical protein |
| *HMPREF0424_1257* | Unknown | Uncharacterized protein family | Hypothetical protein |
| *HMPREF0424_0868* | Cytoplasmatic | Possible lysine decarboxylase | Rossman fold protein, TIGR00730 family |
| *HMPREF0424_0377* | Unknown | YceG-like family | Hypothetical protein |
| *HMPREF0424_0567* | Unknown | Uncharacterized protein family | Hypothetical protein |
| *HMPREF0424_0460* | Cytoplasmatic membrane | ABC-type cobalt transport system, permease component | Hypothetical protein |
| *HMPREF0424_1007* | Cytoplasmatic membrane | EamA-like transporter family | Transporter |
| *HMPREF0424_0833* | Cytoplasmatic membrane | Uncharacterized protein family | Hypothetical protein |
| *HMPREF0424_0912* | Cytoplasmatic | Uncharacterized protein family | Primosome assembly protein PriA |
| *HMPREF0424_0219* | Cytoplasmatic membrane | Uncharacterized protein family | Hypothetical protein |
| *HMPREF0424_0230* | Unknown | Uncharacterized protein family | Hypothetical protein |
| *HMPREF0424_1136* | Cytoplasmatic membrane | Uncharacterized protein family | Membrane protein |
| *HMPREF0424_0378* | Cytoplasmatic membrane | Uncharacterized protein family | Hypothetical protein/peptidase A24 |
| *HMPREF0424_0851* | Cytoplasmatic membrane | Uncharacterized protein family | Membrane protein |
| *HMPREF0424_0301* | Cytoplasmatic membrane | Uncharacterized conserved protein | Hypothetical protein |
| *HMPREF0424_0119* | Unknown | Uncharacterized protein family | ATPase |
| *HMPREF0424_0186* | Cytoplasmatic membrane | Acyltransferase family | Acyltransferase |
| *HMPREF0424_1130* | Cytoplasmatic membrane | Domain of unknown function | Hypothetical protein |
| *HMPREF0424_0162* | Cytoplasmatic membrane | Domain of unknown function, predicted membrane protein | Membrane protein |
| *HMPREF0424_0557* | Unknown | Nucleotidyl transferase AbiEii toxin, Type IV TA system | Hypothetical protein |
| *HMPREF0424_1246* | Cytoplasmatic | HD domain, conserved protein domain | Phosphohydrolase |
| *HMPREF0424_1202* | Cytoplasmatic membrane | Uncharacterized protein family | Beta-carotene 15,15'-monooxygenase |
| *HMPREF0424_0231* | Cytoplasmatic membrane | Uncharacterized protein family | Hypothetical protein |
| *HMPREF0424_0713* | Cytoplasmatic | Protein of unknown function | Hypothetical protein |
| *HMPREF0424_1139* | Cytoplasmatic membrane | Domain of unknown function | AI-2E family transporter |
| *HMPREF0424_0016* | Cytoplasmatic membrane | Predicted permease | Permease |
| *HMPREF0424_0939* | Cytoplasmatic | Glycoprotease family | tRNA threonylcarbamoyladenosine biosynthesis |
| *HMPREF0424_0015* | Cytoplasmatic membrane | Uncharacterized protein family | Membrane protein |
| *HMPREF0424_0792* | Cytoplasmatic membrane | Uncharacterized protein family | Hypothetical protein |
| *HMPREF0424_0579* | Unknown | Uncharacterized protein family | Hypothetical protein |
| *HMPREF0424_0352* | Cytoplasmatic membrane | Uncharacterized protein family | Hypothetical protein / ABC transporter |
| *HMPREF0424_1154* | Cytoplasmatic membrane | UvrD-like helicase C-terminal domain | Helicase |
| *HMPREF0424_0170* | Cytoplasmatic membrane | Uncharacterized protein family | Histidine kinase |
| *HMPREF0424_0823* | Cytoplasmatic membrane | Protein of unknown function | Membrane protein |
| *HMPREF0424_0719* | Cytoplasmatic membrane | Uncharacterized protein family | Hypothetical protein |
| *HMPREF0424_0158* | Cytoplasmic membrane | ABC-2 family transporter protein | hypothetical protein |
| *HMPREF0424_1108* | Unknown | Uncharacterized protein family | hypothetical protein |
| *HMPREF0424_0799* | Cytoplasmatic membrane | Bacterial protein of unknown function | Membrane protein |
| *HMPREF0424_0157* | Cytoplasmatic membrane | ABC-2 family transporter protein | Lantibiotic ABC transporter permease |
| *HMPREF0424_0229* | Cytoplasmatic | Uncharacterized protein family | Helicase |
| *HMPREF0424_1301* | Cytoplasmatic membrane | Protein of unknown function, DUF624 | Beta-carotene 15,15'-monooxygenase |
| *HMPREF0424_0249* | Cytoplasmatic | Uncharacterized protein family | IMPACT family protein |
| *HMPREF0424_0418* | Unknown | Uncharacterized protein family | Hypothetical protein |
| *HMPREF0424_0592* | Cytoplasmatic | Zinicin-like metallopeptidase | Peptidase |
| *HMPREF0424_0909* | Extracellular | WhiA N-terminal LAGLIDADG-like domain | DNA-binding protein WhiA |
| *HMPREF0424_0200* | Cytoplasmatic | Uncharacterized protein family | Hypothetical protein |
| *HMPREF0424_0801* | Unknown | Bacterial protein of unknown function | Hypothetical protein |
| *HMPREF0424_0208* | Unknown | Uncharacterized protein family | Hypothetical protein |
| *HMPREF0424_0283* | Cytoplasmic | Uncharacterized protein family | Hypothetical protein |
| *HMPREF0424_0727* | Extracellular | Uncharacterized protein family | DNA methyltransferase |
| *HMPREF0424_0505* | Cytoplasmatic membrane | Uncharacterized protein family | Exodeoxyribonuclease V |
| *HMPREF0424_0857* | Cytoplasmatic membrane | UPF0126 domain | Membrane protein |
| *HMPREF0424_1293* | Cytoplasmatic membrane | TraX protein | Endonuclease VII |
| *HMPREF0424_0595* | Cytoplasmatic membrane | Uncharacterized protein family | Hypothetical protein |
| *HMPREF0424_0389* | Cytoplasmatic | Protein of unknown function | Hypothetical protein |
| *HMPREF0424_0591* | Unknown | Protein of unknown function | Hypothetical protein |
| *HMPREF0424_0325* | Cytoplasmatic | KH domain | RNA-binding protein |
| *HMPREF0424_0159* | Cytoplasmatic | Type I restriction and modification enzyme - subunit R C terminal | Hypothetical protein |
| *HMPREF0424_1160* | Cytoplasmatic membrane | Iron permease FTR1 family | Iron permease |
| *HMPREF0424_1037* | Cytoplasmatic membrane | Uncharacterized conserved protein | Hypothetical protein |
| *HMPREF0424_0818* | Cytoplasmatic membrane | Uncharacterized conserved protein | Beta-carotene 15,15'-monooxygenase |
| *HMPREF0424_0250* | Unknown | Uncharacterized conserved protein | AbrB family transcriptional regulator |
| *HMPREF0424_1150* | Cytoplasmatic | Beta-lactamase superfamily domain | RNase J family beta-CASP ribonuclease |
| *HMPREF0424_0373* | Cytoplasmatic membrane | Uncharacterized conserved protein | Hypothetical protein |
| *HMPREF0424_1176* | Cytoplasmatic membrane | Uncharacterizsed conserved protein | Hypothetical protein |
| *HMPREF0424_0492* | Cytoplasmatic membrane | Protein of unknown function | Zinc ABC transporter permease |

^a^ All uncharacterized genes encoding hypothetical proteins were up-regulated in biofilms cells

^b^ Protein-protein BLAST (Blastp) results indicated 100% identity with *G. vaginalis*

**Table S3** Primers used in qPCR experiments

| **Target gene** | **Gene description** | **Primer sequence (5’ to 3’)** | **T _melting_ (ºC)** | **Amplicon size (bp)** |
| --- | --- | --- | --- | --- |
| *16S RNA* | 16S ribosomal RNA of *G. vaginalis* | Fw TGAGTAATGCGTGACCAACC  Rv AGCCTAGGTGGGCCATTACC | 55.2  59.3 | 167 |
| *HMPREF0424_0103* (*vly*) | Thiol-activated cytolysin vaginolysin | Fw GAACAGCTGGGCTAGAGGTG  Rv AATTCCATCGCATTCTCCAG | 60.01  60.04 | 153 |
| *HMPREF0424_0471 (gap)* | Glyceraldehyde 3-phosphate dehydrogenase domain-containing protein | Fw AAGAACCAGCGGAAACAATG  Rv ATGGCGTTGAATTCGTTCTC | 60.11  60.08 | 192 |
| *HMPREF0424_0343 (pgi)* | Gucose-6-phosphate isomerase | Fw ATCGCGTGGATAAGTTGAGC  Rv TGCAAAACTGCACGATCTTC | 60.24  60.00 | 184 |
| *HMPREF0424_1220* | Aspartate transaminase | Fw TCGTCAAGCAACATTTCAGC  Rv TAGACGCAAAGCAATTGTGG | 60.00  59.87 | 174 |
| *HMPREF0424_0125* | TadE-like protein | Fw GGTTCTGGCACTATGCTTGG  Rv ACACGCATTATCCTCCATCC | 58.90  57.45 | 171 |
| *HMPREF0424_0821* | Glycosyltransferase, group 2 family protein | Fw CAACGAAGGCATAGGTTTCC  Rv GCGCTTGGAACTGCTTTAAC | 59.57  60.02 | 156 |
| *HMPREF0424_1336* | Periplasmic binding protein and sugar binding domain of the LacI family protein | Fw ATGGCACCTAATGCCATCTC  Rv GGCAAAGGATTCAAAGATCG | 59.92  59.65 | 173 |
| *HMPREF0424_1286 (thiO)* | Glycine oxidase | Fw AATGCCGTGACGGAAGTAAC  Rv ATGACCGCGATATTCCAAAG | 60.00  59.92 | 200 |
| *HMPREF0424_1122* | Multidrug resistance ABC transporter | Fw CAGCACCTGTAGCTCCAACA  Rv TGGCTCAAGAGATTGTGTGC | 60.05  59.99 | 195 |
| *HMPREF0424_0156* | Bacitracin transport ATP-binding protein BcrA | Fw CCGACCGCATACCTATTTTG  Rv GCAAGACGGTCTCCAAACTC | 60.34  59.85 | 178 |
| *HMPREF0424_0354* | Drug resistance MFS transporter | Fw AACCAAGCAATTCCACAAGC  Rv CCGTCGTTTTGGCAGTATTT | 60.12  60.00 | 199 |
| *HMPREF0424_1196* | LPXTG-motif cell wall anchor domain-containing protein | Fw TGCAAAGACAGGCGATAGTG  Rv TAATCGTTGCGGTTGTTTCA | 60.00  60.11 | 173 |

|  |  |  |  |  |
| --- | --- | --- | --- | --- |

**Supplementary figures legends**

**Fig. S1.** Gene interaction network generated using Cytoscape, showing down-regulated transcripts (fold-change ≤ -2) in red and up-regulated transcripts (fold-change ≥ 2) in green. Yellow circles correspond to transcripts differentially expressed with a fold-change between -2 and 2.

## Fig. S2. Glycolysis/gluconeogenesis pathway of *G. vaginalis* 409-05 by KEGG Pathway Maps, available at: http://www.genome.jp/kegg-bin/show_pathway?map00010. Green boxes correspond the known pathways in *G. vaginalis* 409-05. Down-regulated transcripts (fold-change ≤ -2) are represented in red. Yellow circles correspond to transcripts differentially expressed with a fold-change between -2 and 2. The genes responsible in the pathways 2.7.23 (*pgk*) and 1.1.1.27 (*HMPREF0424_0663*) are annotated in *G. vaginalis* 409-05, but they were not transcribed in our experiments.
